# A novel biomechanical system to intrude the upper incisors and control overbite: Posterior miniscrew-assisted lever arm and 2 cases report

**DOI:** 10.1097/MD.0000000000031616

**Published:** 2022-11-25

**Authors:** Chenghao Zhang, Ling Ji, Wen Liao, Zhihe Zhao

**Affiliations:** a Department of Orthodontics, West China Hospital of Stomatology, State Key Laboratory of Oral Diseases & National Clinical Research Center for Oral Diseases, Sichuan University, Chengdu, China; b West China Hospital of Stomatology, State Key Laboratory of Oral Diseases & National Clinical Research Center for Oral Diseases, Sichuan University, Chengdu, China.

**Keywords:** case report, incisor intrusion, miniscrews, overbite

## Abstract

Overbite control is a key factor in orthodontic treatment. In some cases, incisor intrusion is essential and could be an optimal strategy for overbite control. The aim of this article was to introduce a biomechanical system called the posterior miniscrew-assisted lever arm, which is innovative in using existing posterior miniscrews to intrude the upper incisors and to control anterior overbite while simultaneously retracting the anterior teeth. Its efficiency in incisor intrusion has been proved with 2 cases.

**Patient concerns:**

Two adult women who came for orthodontic treatment with the chief complaint of convex profile were included in this study.

**Diagnosis:**

Both patients had similar malocclusions of Class II molar relationship, anterior deep overjet, and anterior deep overbite.

**Interventions:**

Their treatment plans were to extract 4 first premolars and insert 2 maxillary posterior buccal miniscrews. After teeth aligning and leveling, en masse retraction was started in both arches. During the space-closing stage, posterior miniscrew-assisted lever arms were placed in their upper arches so as to intrude upper incisors and control the overbite.

**Outcomes:**

After respectively 4 months and 3 months of incisor intrusion, the anterior overbite was successfully reduced to the normal range in each patient. Cephalometric analysis and superimposition also confirmed the treatment effect of this biomechanical system on incisor intrusion.

**Lessons:**

The posterior miniscrew-assisted lever arm is a valuable biomechanical system for intruding incisors and controlling anterior overbite.

## 1. Introduction

Facial esthetic improvement is one of the most common reasons for seeking orthodontic treatment.^[1]^ For patients with chief complaints of convex profile and/or protrusive teeth, their malocclusion types could be in Angle Class II Division 1 malocclusions with protrusive maxillary dentition or Class I malocclusions with bimaxillary dentoalveolar protrusion.^[2]^ Premolar extraction is normally planned for their treatment and anchorage control is essential for a successful treatment effect. Although premolar extraction sequences can be different, it is believed that compared with second premolars extraction, first premolars extraction has a better effect on retracting the incisors and the upper and lower lips, increasing the nasolabial angel, and thus improving the facial profile esthetics.^[3,4]^ Moreover, to resist the undesirable molar mesial movement and to retract the anterior teeth as much as possible, posterior miniscrew is wildly used for anchorage reinforcement.^[5,6]^

Posterior miniscrews can provide stable skeletal anchorage and overcome problems of anchorage loss during extraction space closure. It can be simultaneously used with various orthodontic techniques, such as sliding mechanics and loop mechanics, and has proved efficacy in anchorage control in the anteroposterior and vertical directions.^[7–9]^ In the anteroposterior direction, posterior miniscrews have been frequently used to re-tract the anterior teeth or distalise the molars.^[10,11]^ To facilitate these types of tooth movement, the miniscrews are directly applied to archwire hooks with elastics or li-gated with the premolars or molars as an indirect anchorage.^[12]^ In vertical direction, posterior miniscrews can help intruding the molars as direct anchorage, which is beneficial for the treatment of open bite malocclusion.^[13]^ Using auxiliary devices that are connected with posterior miniscrews, the molars can also be extruded, which helps correct occlusal-plane canting.^[14]^ However, when it comes to anterior teeth, using posterior miniscrews alone can hardly provide anchorage in vertical dimension, which poses the need for extra anchorage techniques to intrude the anterior teeth.

Therefore, this article develops a biomechanical system called “posterior minis-crew- assisted lever arm,” with which we use the existed posterior miniscrews to intrude the upper incisors and control anterior overbite during orthodontic treatment. It consists of 2 posterior buccal miniscrews already inserted in the upper arch and 1 auxiliary lever arm. With the system, the upper incisors could be easily and efficiently intruded and the treatment time could be reduced.

## 2. Posterior miniscrew-assisted lever arm

**Figure 1.**
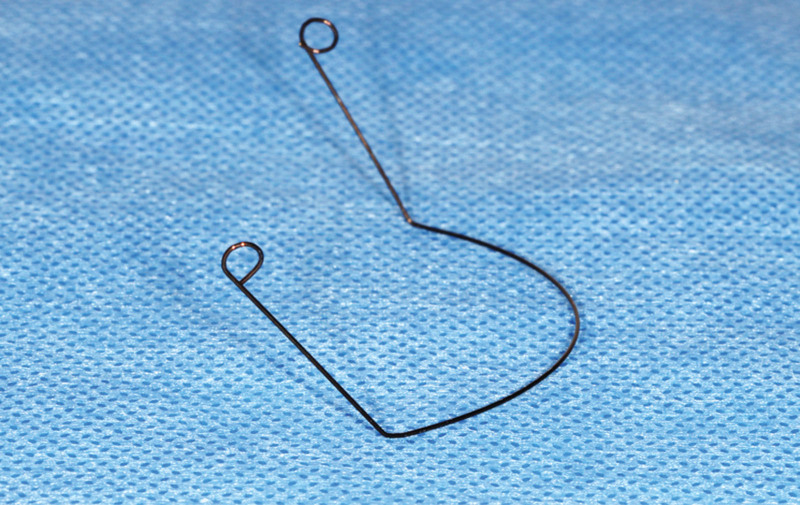
The intrusion lever arm is formed with a 0.016-inch stainless steel round wire (Australian Wire).

**Figure 2.**
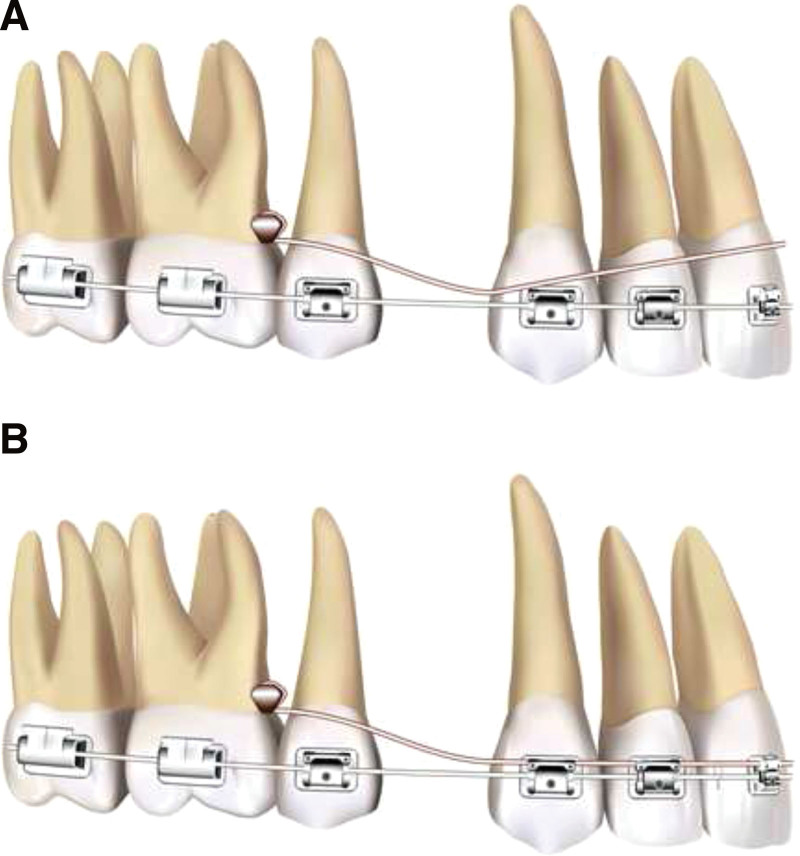
Schematic diagrams of the usage of posterior miniscrew-assisted lever arm. (A) Before placement. (B) After ligating the anterior part of lever arm to the main arch wire, the biomechanical system is activated.

In premolars extraction cases with skeletal anchorage, the upper miniscrews are normally inserted in the maxillary buccal alveolar bone, between the roots of the second premolar and the first molar or between the first and second molar.^[15]^ The intrusion lever arm is formed with a 0.016-inch stainless steel round wire (Australian Wire) of appropriate length with hook on each end for engagement to the head of miniscrew (Fig. 1). There is a bend which is distal to the canine’s bracket on each side making the anterior part curve to the gingival side. After ligating the anterior part of the lever arm to the main arch wire, this biomechanical system is activated and the upper incisors will be intruded (Fig. 2). The degree of bend, the size of lever arm, and the force of anterior ligation can be adjusted in accord with the need for the intrusive force.

## 3. Case presentation

Two adult women who came for orthodontic treatment with the chief complaint of convex profile were included in this study. They both had initial deep anterior overjet and overbite, with bilateral Class II molar and canine relationship. After analyzing their clinical examination findings and records, we decided a nonsurgical extraction treatment plan for each of them. The 4 first premolars were extracted; 2 maxillary posterior miniscrews were placed in the buccal alveolar bone between the roots of the maxillary second premolar and first molar to help retract the upper anterior teeth, correct the molar and canine relationships and improve the soft tissue profile.

After normally brackets bonding, teeth aligning and leveling, en masse retraction was started. To counter the side effects of sliding mechanics and Class II elastics and to control the anterior overbite, a posterior miniscrew-assisted lever arm was placed in the upper arch and the upper incisors would be intruded. The en masse retraction and Class II elastics continued during the incisors’ intrusion.

**Figure 3.**
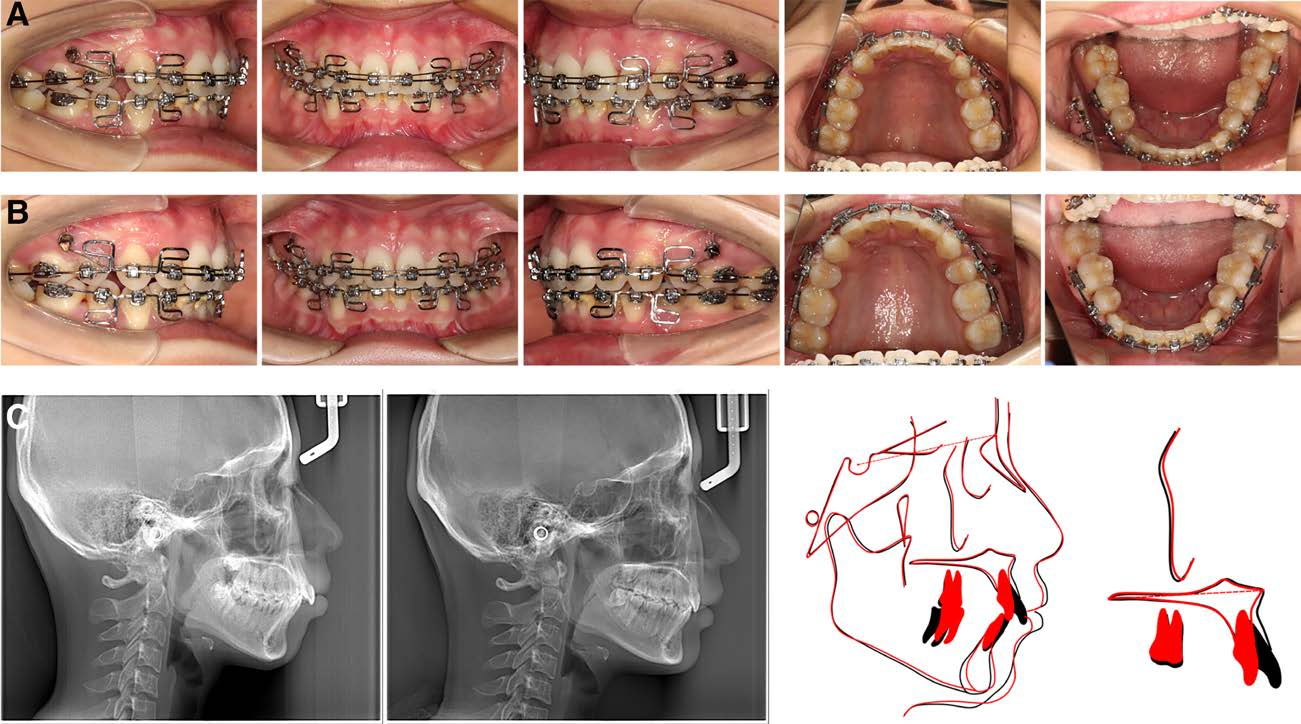
The intraoral features and cephalometric superimposition of patient 1. (A) The intraoral features of patient 1 before incisors intrusion. (B) The intraoral features of patient 1 after incisors intrusion. (C) Pretreatment and post-treatment cephalometric radiographs and superimposed tracings of patient 1: black line, pretreatment; red line, post-treatment. The upper incisor was apparently intruded.

**Figure 4.**
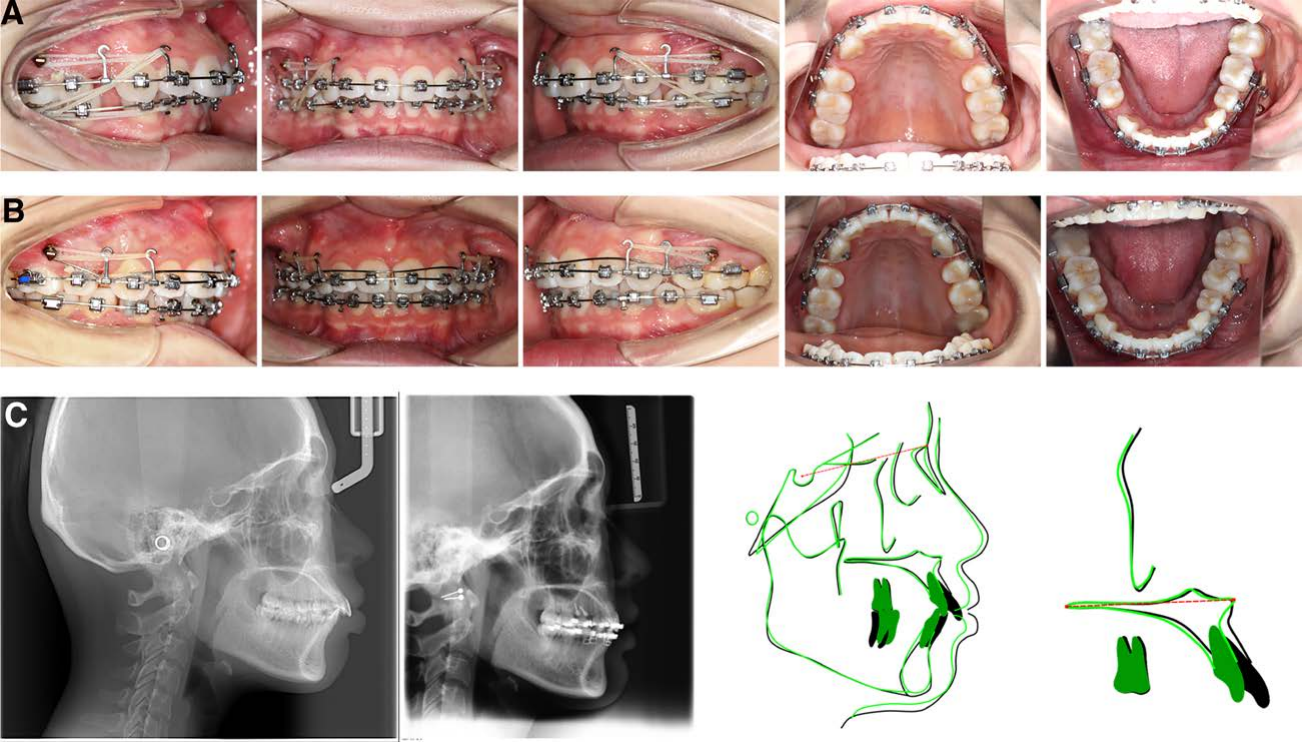
The intraoral features and cephalometric superimposition of patient 2. (A) The intraoral features of patient 2 before incisors intrusion. (B) The intraoral features of patient 2 after incisors intrusion. (C) pretreatment and post-treatment cephalometric radiographs and superimposed tracings of patient 2: black line, pretreatment; green line, after incisor intrusion. The upper incisor was apparently intruded.

After 4 months and 3 months of lever arm application respectively, the overbite was successfully reduced to the normal range in each patient. Finishing and detailing of the occlusion was then performed. The cephalometric analysis and superimposition indicted that the upper incisors of both patients were apparently intruded (Figs. 3 and 4). Compared with the pretreatment, the value of U1-PP decreased about 1.3 and 1.9 mm, respectively (Tables 1 and 2).

**Table 1:** Cephalometric analysis of patient 1.

	Pre-treatment	Post-treatment	Norm (±SD)
SNA (°)	83.7	83.2	83.0 ± 4.0
SNB (°)	76.1	76.6	80.0 ± 4.0
ANB (°)	7.6	6.6	3.0 ± 2.0
MP-FH (°)	29.0	26.3	26.0 ± 4.0
SN—MP (°)	37.0	33.3	30.0 ± 6.0
U1-PP (mm)	30.7	29.4	28.0 ± 2.0

ANB = the angle between N-A plane and N-B plane, MP-FH = the angle between Mandibular plane and F-H (Frankfort horizontal) plane, SNA = the angle between S-N plane and N-A plane, SNB = the angle between S-N plane and N-B plane, SN-MP = the angle between S-N plane and Mandibular plane, U1-PP = the distance from Upper 1 to P-P (ANS-PNS) plane.

**Table 2:** Cephalometric analysis of patient 2.

	Pre-treatment	Post-treatment	Norm (±SD)
SNA (°)	81.3	79.4	83.0 ± 4.0
SNB (°)	76.6	76.9	80.0 ± 4.0
ANB (°)	4.7	2.5	3.0 ± 2.0
MP-FH (°)	20.0	20.5	26.0 ± 4.0
SN—MP (°)	31.3	31.7	30.0 ± 6.0
U1-PP (mm)	30.5	28.6	28.0 ± 2.0

ANB = the angle between N-A plane and N-B plane, MP-FH = the angle between Mandibular plane and F-H (Frankfort horizontal) plane, SNA = the angle between S-N plane and N-A plane, SNB = the angle between S-N plane and N-B plane, SN-MP = the angle between S-N plane and Mandibular plane, U1-PP = the distance from Upper 1 to P-P (ANS-PNS) plane.

## 4. Discussion

The system we introduced here was called posterior miniscrews-assisted lever arm. Its efficiency in intruding the upper incisors is supported by intraoral photographs and cephalometric superimposition in the 2 cases. The crown of upper central incisor was intruded 1.3mm in patient 1 and 1.9mm in patient 2 according to the value of U1-PP (mm). Considering the torque of upper incisor is decreased, the true intrusion amount of the root should be more. Besides, the values of the angle between sella-nasion plane and mandibular plane (°) and FH-MP (°) decreased after treatment in patient 1 and well-controlled in patient 2, which indicated that the bite-opening is not caused by posterior teeth elongation but incisors intrusion. Especially in patient 1, the mandibular angle decreased suggested mandibular counter-clockwise rotation, which was beneficial for correction of Skeletal Class II malocclusion.

**Figure 5.**
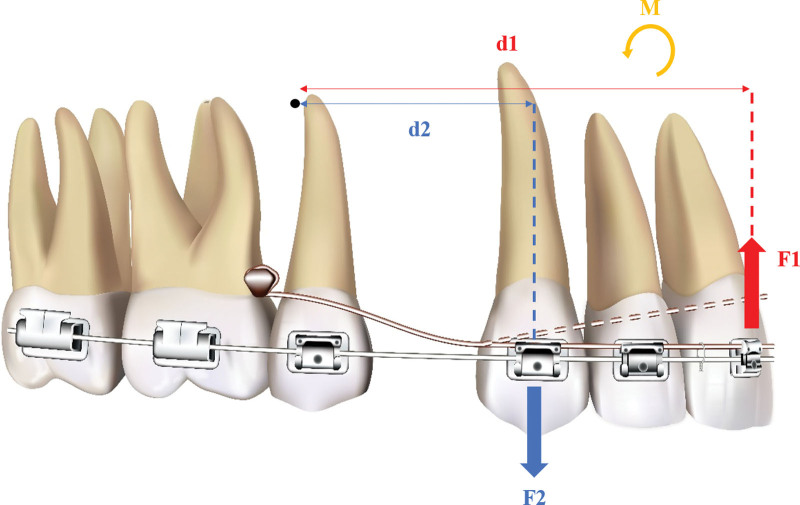
Schematic diagram of force analysis of the biomechanics system. F1, the intrusive force on the upper incisors; F2, the extrusive force on the canine; d1, the arm of F1; d2, the arm of F2; M, the counterclockwise rotation moment.


The force analysis manifests how this system has an effect on bite-opening and torque control (Fig. 5). After activating this lever arm by ligating its anterior part to the main arch wire, the resilience force of the lever arm becomes the intrusive force on the upper incisors (F1). After the intrusion, the anterior overbite is decreased. Meanwhile, the canine gives lever arm a supportive force so the lever arm also has an extrusive force act on the canine (F2). The intrusive force of incisor and the extrusive force of canine give maxillary dentition a counterclockwise rotation moment that causes the upper incisors to be labially inclined and improves torque control in extraction cases. The center of resistance of maxillary dentition is near the apical root of maxillary second premolar; after first premolar extraction, the center of resistance is expected to move distally. The arm of F1 is d1, whose length is the perpendicular line from F1 to the center of resistance, and the arm of F2 is d2. The value of this moment can be summarized with the following formula:

$$\begin{array}{l} M=F1*d1-F2*d2 \end{array}$$

Because the lever arm is formed with Australian wire, which has high resiliency and toughness, the value of F1 is bigger than F2. As F1 and F2 are both at the mesial side to the center of resistance, the center of rotation is at the distal side to the center of resistance. Hence, the molars would be rarely affected by this moment.

Another advantage of this system is that it is time-saving in that the retraction elastics can be simultaneously used to close the residual space while using the lever arm. The lever arm is ligated occlusal to the main arch wire with little friction force countering the retraction force. Hook on each end is placed on the head of miniscrews, which is small and would not interfere the placement of coil spring or elastomeric power chain. Besides, even Class II elastics needs to be used to correct the molar relationship, the intrusive force of incisors and the counterclockwise moment can be regulated flexibly to counter the side effects caused by the elastic force by changing the degree of bend, the size of lever arm, or the force of anterior ligation.

There are many reported methods about intruding upper incisors to correct anterior deep overbite in extraction cases. One popular method is to use anterior miniscrew inserted between the roots of anterior teeth to intrude the incisors.^[16]^ It can be 1 miniscrew inserted between the upper central incisors or 2 miniscrews inserted between the upper lateral incisor and canine. The method has been demonstrated to be efficient in deep overbite correction no matter with 1 or 2 miniscrews.^[17]^ However, the usage of miniscrew is accompanied with multiple adverse effects, including biologic damage, inflammation, pain and discomfort.^[18,19]^ With this method, at least 1 extra miniscrew needs to be inserted into maxillary anterior alveolar bone, which would increase the risk and cost and turn out to be not friendly enough for patients.

Another popular method is to choose optimal insertion height and position of posterior miniscrews and use long traction hooks to change the direction of retraction force.^[20–22]^ Theoretically, an optimal combination between the position of posterior miniscrews and the length of traction hooks can greatly help control the change of overbite and anterior torque. However, the structure relationship of alveolar bone and incisors is different in each patient, which makes it difficult to locate the center of resistance and control the force direction. It also has a high requirement for clinical experience to figure out the optimal combination and to precisely miniscrews into the exact area. Besides, the vestibular sulcus depth or alveolar bone areas of some patients may not enough to support miniscrews inserted at the needed height.^[23]^

There is also a classical method that uses utility arch, a conventional auxiliary arch whose effects on intruding incisors are similar with that of anterior miniscrews.^[24,25]^ However, the traditional utility arch is a segmented arch wire with which the incisor intrusion and dentition aligning could not be applied at the same time. This would prolong the treatment duration and reduce patient satisfaction. Besides, even the utility arch is used as an auxiliary wire and would not interfere dentition aligning and space closing, this method has several side effects that may cause treatment failure, such as causing uncontrolled molar’s extrusion and distally rotation, and incisors being labially inclined rather than truly intruded.^[26,27]^

Taken all aspects into consideration, this biomechanical system is innovative in the way it uses the existed posterior miniscrews to intrude the upper incisors without any other additional injury risk or side effects. The 2 clinical cases demonstrate the system’s efficiency in intruding the upper incisors. What’s more, in theory, this system has the potentiality to be applied in mandibular arch to intrude the lower incisors.

## 5. Conclusion

The posterior miniscrew-assisted lever arm is a valuable biomechanical system for intruding incisors and controlling anterior overbite. It has proved efficiency in bite-opening by this work.

## Author contributions

Chenghao Zhang participated in the clinical treatment of the patients, data analysis, and was responsible for the literature search and wrote the paper. Ling Ji participated in data collection and schematic diagram drawing. Wen Liao and Zhihe Zhao revised and edited the manuscript and figures. All authors read and approved the final manuscript.

**Data curation:** Chenghao Zhang, Ling Ji.

**Investigation:** Chenghao Zhang.

**Resources:** Ling Ji.

**Supervision:** Wen Liao, Zhihe Zhao.

**Writing – original draft:** Chenghao Zhang.

**Writing – review & editing:** Wen Liao, Zhihe Zhao.
